# Improving Smartphone GNSS Positioning Accuracy Using Contextual Information

**DOI:** 10.3390/s26113346

**Published:** 2026-05-25

**Authors:** Bong-Gyu Park, Jong-Sung Lee, Miso Kim, Kwan-Dong Park

**Affiliations:** 1 Department of Geoinformatic Engineering, Inha University, 100 Inha-ro, Incheon 22212, Republic of Korea; geek1206@inha.edu (B.-G.P.); jslee99@inha.edu (J.-S.L.); mskim@ppsol.com (M.K.); 2 PPSOL Inc., 606 Seobusaet-gil #B-2311, Seoul 08504, Republic of Korea

**Keywords:** smartphone, GNSS, RTK, ECI, Kalman filter, adaptive positioning, adaptive Kalman filtering

## Abstract

With the widespread adoption of smartphones, location-based services have become increasingly important. Consequently, accurate and reliable global satellite navigation system positioning on smartphones has become essential. However, achieving accurate positioning in urban areas remains challenging because of the inherent limitations of smartphones and severe multipath effects. To address this issue, this study proposes two methods to improve positioning accuracy using contextual information. First, an environmental context indicator was used to refine the C/N_0_-based observation covariance model. Second, normalized C/N_0_ and code-pseudorange residuals were used to detect non-line-of-sight satellites and adjust the observation covariance. Experiments were conducted in both open and urban areas, and performance was evaluated using circular error probable (CEP) and distance root mean square (DRMS). The experimental results showed that, in open areas, the proposed method achieved submeter to decimeter-level horizontal accuracy and precision. In semi-urban areas, CEP95, CEP50, and DRMS decreased by approximately 8, 2, and 4 m, respectively. In urban canyons, CEP95, CEP50, and DRMS decreased by approximately 15, 2, and 5 m, respectively.

## 1. Introduction

With the widespread adoption of smartphones, location-based services (LBS) have attracted considerable attention in modern society. They are used for vehicle and pedestrian navigation, as well as in applications such as gaming, healthcare, and logistics [1]. Realizing these services requires multiple technologies, including global navigation satellite systems (GNSS), Wi-Fi, and digital maps. Among these, GNSS is used to determine user location, and smartphone GNSS positioning has become a key technology for enabling LBS [2].

Research on smartphone GNSS has expanded since the release of Android OS 7.0, which enabled access to GNSS raw measurements. Compared with conventional GNSS receivers, smartphones generally exhibit lower observation quality because of their linearly polarized antennas, which are more susceptible to signal attenuation [3,4]. In urban areas, the observation quality is further degraded by severe multipath and cycle slips [5,6]. Consequently, position estimates obtained from smartphone GNSS are often less accurate. Therefore, further research is required to improve smartphone GNSS positioning accuracy.

Despite these limitations, many studies have proposed approaches to improve smartphone GNSS positioning accuracy. Existing studies are broadly classified into four categories: (1) multi-constellation and dual-frequency positioning, (2) outlier detection and stochastic modeling, (3) 3D model-aided positioning, and (4) context-aware positioning. The first category improves positioning performance by increasing the number of available satellites and enhancing observation quality. The second category improves robustness by mitigating outliers and refining the stochastic model. The third category utilizes resources from 3D databases, and the fourth category applies observation environment classification to improve positioning accuracy. Major studies in each area are reviewed in the following paragraphs.

First, multi-constellation and dual-frequency positioning are widely used approaches for improving smartphone GNSS positioning accuracy. This approach improves positioning accuracy by using multiple GNSS constellations and enhancing satellite geometry, and the use of L5 signals further improves accuracy because they are more robust to multipaths than L1 signals. Several studies have demonstrated the efficacy of these methods. Robustelli et al. [7] performed standard point positioning using a Xiaomi Mi 8 smartphone and showed that, compared with the use of Global Positioning System (GPS) alone, the use of GPS, Global Navigation Satellite System (GLONASS), and Galileo can reduce horizontal error by more than 1 m. Moreover, they reported that L5 observations exhibit less than half the multipath errors of L1 observations. Yun and Park [8] showed that the use of L5 observations improves differential GNSS positioning accuracy by several meters.

Second, outlier detection and stochastic modeling improve positioning accuracy by mitigating the effects of outliers and enhancing stochastic models using additional factors. In urban areas, observation quality is inevitably degraded by surrounding buildings and obstacles. Consequently, robust weighting schemes such as IGG-III can be used to mitigate the impact of large observation residuals by adjusting their weights [9]. Such weighting schemes have been reported to suppress the divergence of position estimates and improve positioning accuracy [10,11]. Meanwhile, modeling an appropriate observation covariance is an important step toward improving positioning accuracy. Observation residuals are highly correlated with C/N_0_ [12,13]. Therefore, a C/N_0_-based stochastic model, known as the SIGMA model, has been widely used. However, because the SIGMA model is sensitive to changes in the observation environment, Zangenehnejad and Gao [14] proposed a new stochastic model that combines C/N_0_ and elevation angles. They demonstrated that the combined model improves horizontal accuracy by approximately 25%. In addition, Hu et al. [15] found that positioning accuracy varies depending on the carrier-to-code noise ratio in the observation covariance model. They demonstrated that an adaptive carrier-to-code ratio is more effective than a fixed ratio. Moreover, velocity estimation can be used to enhance stochastic modeling [16]. These studies show that traditional stochastic models require further refinement to improve positioning performance.

Third, 3D model-aided positioning differs from the techniques applied in existing studies because it relies on an external 3D database. A representative example is shadow matching (SM), which adjusts position estimates using a building database in urban environments such as urban canyons [17]. In previous studies, smartphone NMEA data were used to apply SM because raw GNSS data were inaccessible [18]. Subsequently, a robust SM that uses machine learning techniques was proposed and shown to improve smartphone positioning accuracy in urban areas [19]. A key advantage of this approach is that it can estimate both line-of-sight (LOS) and non-line-of-sight (NLOS) satellites in urban environments.

Finally, context-aware positioning classifies the observation environment and uses the classification result as an adaptive factor to improve positioning accuracy under varying environmental conditions. Environmental context is categorized into several classes based on observation residuals, C/N_0_, the number of visible satellites, and position dilution of precision (PDOP). In existing studies, hidden Markov models and fuzzy inference systems were proposed to classify open-sky, urban, intermediate, and indoor environments [20]. Subsequently, machine learning models and neural networks were proposed to classify various types of environments [21,22,23,24]. Meanwhile, several studies have reported that the observation environment can be distinguished using metrics derived from raw GNSS data [25,26,27]. Park et al. [27] proposed an environmental context indicator (ECI), which is calculated using C/N_0_ and PDOP. ECI comprises a float indicator (ECI-F), an integer indicator (ECI-I), and an uncertainty indicator corresponding to ECI-I. The ECI suggests that environmental context can take various forms when evaluating environmental conditions. Despite a wide range of studies, only a few have applied the environmental context to improve positioning accuracy. Zhu et al. [24] improved the horizontal accuracy of u-blox receivers by approximately 18% using a strategy that adjusts the C/N_0_-based stochastic model in response to changes in environmental context. However, further research is required on context-aware positioning because methods for incorporating contextual information into positioning models are still underdeveloped.

In summary, multi-constellation and dual-frequency positioning are important for improving smartphone GNSS positioning accuracy, whereas outlier detection and stochastic modeling are essential for robust estimation. However, 3D model-aided approaches cannot easily be applied in areas where 3D databases are unavailable, and their effectiveness remains limited unless the databases are regularly updated. In context-aware positioning, refining traditional stochastic models using categorical information is also limited because a few classes may not adequately represent continuously varying environmental conditions. Moreover, classification accuracy can decrease in urban areas, where buildings and obstacles are densely distributed. Therefore, further studies are required to develop a method that can improve positioning accuracy while simultaneously describing environmental conditions.

To address this research gap, this study proposes methods to improve the conventional C/N_0_-based stochastic model by incorporating environmental context information and NLOS satellite detection. These methods are intended to enhance the adaptability and robustness of the positioning filters. First, we performed real-time kinematic (RTK) positioning and quantified contextual information using ECI-F. Second, we characterized the traditional C/N_0_-based stochastic model and derived a strategy for its refinement. Third, we constructed a regression model to inflate the observation covariance and utilized normalized C/N_0_ and code-pseudorange residuals to detect NLOS satellites, thereby mitigating the effects of outliers.

The remainder of this paper is organized as follows: Section 2 introduces the data acquisition process and RTK implementation. Section 3 describes the conventional C/N_0_-based stochastic model and discusses possible improvements. Section 4 presents an adaptive observation covariance model based on ECI and NLOS satellite detection. Section 5 discusses the key findings and limitations of the study. Finally, Section 6 provides a summary and concluding remarks.

## 2. Materials and Methods

This section summarizes the data collection, smartphone RTK implementation, and stochastic model used in the adaptive covariance scheme.

### 2.1. Data Acquisition

A Samsung Galaxy S21+ smartphone was used for data collection. As shown in Table 1, the device supports multi-constellation, including a dual-frequency GPS/Quasi-Zenith Satellite System and Galileo. In addition, an in-house logger was used to record raw GNSS measurements for real-time processing.

For RTK implementation, GPS, Galileo, and BeiDou were used, whereas GLONASS was excluded because its code-pseudorange quality was inferior to that of the other systems. In addition, a satellite-based augmentation system (SBAS) was not required for RTK since the short-baseline double-differenced RTK model substantially reduces the major common errors, including satellite clock, receiver clock, orbital, and atmospheric errors. Similarly, Navigation with Indian Constellation (NavIC) and QZSS were excluded because they contribute only a limited number of visible satellites. During data collection, duty cycling was disabled to avoid carrier -phase discontinuities.

**Figure 1:**
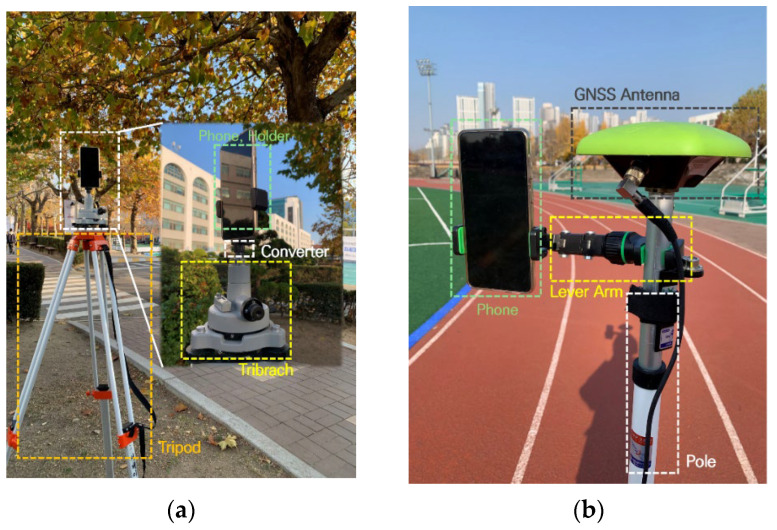
Experimental setups for smartphone and reference data acquisition: (**a**) static data acquisition; (**b**) kinematic data acquisition.

Figure 1 shows the experimental setup. We mounted the smartphone on a tripod during static data collection and placed it on a pole with a lever arm for kinematic data collection. A Javad G5T antenna for reference data collection and a smartphone were mounted together on the pole, allowing simultaneous data collection.

Reference coordinates were obtained using an RTAP2U receiver [28] from PPSOL Inc. (Seoul, Republic of Korea) and the virtual reference station service of the National Geographic Information Institute. The lever-arm offset was not calibrated because centimeter-level accuracy was beyond the scope of the present study.

### 2.2. Implementation of RTK Algorithm

This subsection presents the measurement model and key methods used for RTK implementation.

#### 2.2.1. Measurement Model

Following the observation equations of Misra and Enge [29] and Teunissen and Montenbruck [30], code-pseudorange ($\rho_{r}^{s}\left(t\right)$) and carrier phase ($\Phi_{r}^{s}\left(t\right)$) can be expressed in terms of the geometric range ($r_{r}^{s}\left(t,t-\tau_{r}^{s}\right)$) and error terms. These terms include the receiver and satellite clock offsets ($dt_{r}\left(t\right)$, $dt^{s}\left(t-\tau_{r}^{s}\right)$), hardware delays ($d_{r}^{s}$, $\delta_{r}^{s}$), ionospheric delay ($I_{r}^{s}\left(t\right)$), tropospheric delay ($T_{r}^{s}\left(t\right)$), ambiguity ($N_{r}^{s}\left(t\right)$), and residuals ($\epsilon_{r}^{s}\left(t\right)$), as expressed in Equations (1) and (2) below. In these equations, r, s, c, τ, and λ indicate receiver, satellite, speed of light, signal transmission time, and wavelength, respectively.

(1) $$\rho_{r}^{s}\left(t\right)=r_{r}^{s}\left(t,t-\tau_{r}^{s}\right)+c\left[dt_{r}\left(t\right)+d_{r}^{s}\right]-c\left[dt^{s}\left(t-\tau_{r}^{s}\right)-d^{s}\left(t-\tau_{r}^{s}\right)\right]+I_{r}^{s}\left(t\right)+T_{r}^{s}\left(t\right)+\epsilon_{r}^{s}\left(t\right)\;\;\;[m]$$

(2) $$\Phi_{r}^{s}\left(t\right)=r_{r}^{s}\left(t,t-\tau_{r}^{s}\right)+c\left[dt_{r}\left(t\right)+\delta_{r}^{s}\right]-c\left[dt^{s}\left(t-\tau_{r}^{s}\right)-\delta^{s}\left(t-\tau_{r}^{s}\right)\right]-I_{r}^{s}\left(t\right)+T_{r}^{s}\left(t\right)+\lambda N_{r}^{s}\left(t\right)+\epsilon_{r}^{s}\left(t\right)\;\;\;[m]$$

For RTK, the highest-elevation satellite observed by the rover was selected as the pivot satellite. The observations were first differenced between receivers and subsequently between satellites. As shown in Equations (3) and (4), for short-baseline RTK, common errors in the code observations are cancelled after double differencing, whereas the carrier-phase observations retain double-differenced ambiguities.

(3) $$\rho_{DD}\left(t\right)=\epsilon_{DD,\rho }\left(t\right)\;\;\;[m]$$

(4) $$\Phi_{DD}\left(t\right)=\lambda N_{DD}\left(t\right)+\epsilon_{DD,\Phi }\left(t\right)\;\;\;[m]$$

Therefore, as expressed in Equation (5), the state vector (x) consists of an increment in the rover coordinates and double-difference ambiguities. The observation vector (z) comprises a double-differenced code and carrier-phase observations, and the design matrix (H) contains the line-of-sight geometry in the coordinate columns and the corresponding wavelengths in the carrier-phase ambiguity columns.

(5) $$z=\left[\begin{array}{c} \rho_{DD}^{1}\\ \vdots \\ \begin{array}{c} \rho_{DD}^{n}\\ \begin{array}{c} \Phi_{DD}^{1}\\ \begin{array}{c} \vdots \\ \Phi_{DD}^{n} \end{array} \end{array} \end{array} \end{array}\right],\;H=\left[\begin{array}{cccccc} \partial h_{x}^{1} & \partial h_{y}^{1} & \partial h_{z}^{1} & & & \\ \vdots & \vdots & \vdots & & & \\ \partial h_{x}^{n} & \partial h_{y}^{n} & \partial h_{z}^{n} & & & \\ \partial h_{x}^{1} & \partial h_{y}^{1} & \partial h_{z}^{1} & \lambda^{1} & & \\ \vdots & \vdots & \vdots & & \ddots & \\ \partial h_{x}^{n} & \partial h_{y}^{n} & \partial h_{z}^{n} & & & \lambda^{n} \end{array}\right],\;x=\left[\begin{array}{c} \partial x_{r}\\ \partial y_{r}\\ \partial z_{r}\\ \partial N_{DD}^{1}\\ \vdots \\ \partial N_{DD}^{n} \end{array}\right]$$

#### 2.2.2. Extended Kalman Filtering Strategies

A velocity-aided extended Kalman filter (EKF) was used to improve initial convergence and short-term stability [31,32]. In the prediction step, the rover coordinates were predicted using an external velocity estimate, and the velocity covariance was added, as expressed in Equation (6).

(6) $$\begin{array}{cc} {\hat{x}}_{k}^{-} & =A{\hat{x}}_{k-1}^{-}+V_{r}∆t\\ P_{k}^{-} & =AP_{k-1}A^{T}+Q \end{array}$$

Velocity was computed from time-differenced carrier phase (TDCP) measurements; however, when TDCP was unavailable because of a cycle slip, Doppler-aided velocity was used [33,34]. Velocity was used only as an external aid; therefore, the state-transition matrix was set to the identity matrix, and the ambiguity process noise was modeled as a random walk process.

The filter update step follows the Kalman gain formula, as expressed in Equation (7), where the observation covariance matrix R is the key tuning component.

(7) $$K_{k}=P_{k}^{-}H^{T}{\left(HP_{k}^{-}H^{T}+R\right)}^{-1}$$

For smartphone GNSS, code-pseudorange residuals are known to be more sensitive to C/N_0_ than to elevation angle [12]. Therefore, R was modeled using the SIGMA model in Equation (8), where the variance of each signal is expressed as a function of C/N_0_ and the parameters a and b.

(8) $$\sigma_{i}^{2}=a+b\times 10^{-\left(C/{N_{0}}_{i}\right)/10}$$

The SIGMA model parameters were estimated using double-differenced code-pseudorange from a short-baseline test at Inha University [35]. Code-pseudorange residuals were obtained using the observation-minus-computation value. Next, the residuals were grouped into 0.5 dB-Hz bins, and the standard deviation of the residuals was computed for each bin. The signal-specific parameters were fitted using nonlinear least squares.

As expressed in Equation (9), the carrier-phase covariance was derived from the code-to-carrier noise ratio estimated via triple differencing, which eliminates the ambiguity term when no cycle slip occurs and enables the relative noise level of the code and carrier observations to be estimated [35,36].

(9) $$\frac{\sigma_{\rho }^{2}}{\sigma_{\Phi }^{2}}=\frac{var\left(∆\rho_{br}^{PO}\right)}{var\left(∆\Phi_{br}^{PO}\right)}$$

In general, code-pseudorange residuals are assumed to have meter-level precision, whereas carrier-phase residuals are assumed to have millimeter-level precision. In this study, the parameters listed in Table 2 were adopted as predefined values [35].

In general, a specific variance ratio is applied uniformly to all signal types. However, the ratios estimated from the observations indicate that the variance ratio is signal-dependent. In particular, the estimated ratios for GPS L5 and GAL E5 are lower than those for GPS L1, GAL E1, and BDS B2. These results indicate that a uniform variance ratio may not sufficiently represent the signal-dependent characteristics.

#### 2.2.3. Detecting and Addressing Cycle Slip

Cycle slips frequently occur in smartphone carrier-phase observations because of blockage and tracking interruptions and they must be addressed to maintain consistency of the filter states [37]. The primary detector was the Android Accumulated Delta Range state, which classifies carrier tracking as four states: ‘unknown’, ‘valid’, ‘reset’, or ‘cycle slip’ [38]. Any state other than valid was considered a cycle slip in this study.

In addition, observation-aided detection was considered. Doppler- and code-minus-carrier-based single-frequency methods [39,40] are broadly applicable, whereas dual-frequency methods, such as TurboEdit [41], are less suitable for smartphones because of their large code-pseudorange noise and incomplete dual-frequency support [42]. Therefore, Doppler-aided detection with a threshold of 1.6 cycles was adopted. The threshold was empirically selected conservatively to minimize missed detections, even at the expense of a higher false-detection rate. Because a cycle slip at either the base or rover affects the double-differenced carrier phase observation, both receivers were evaluated. Subsequently, the ambiguity was re-initialized using the code-minus-carrier measurements with an initial covariance of $10^{4}$.

## 3. Adaptive Observation Covariance Model

This section analyzes the characteristics of the SIGMA model in urban environments and the relationship between ECI-F and code-pseudorange residuals. Moreover, we propose two methods for constructing an adaptive observation covariance model. The first method is based on ECI-F, and the second uses normalized C/N_0_ and code-pseudorange residuals. Details are provided in the following subsections.

### 3.1. Characterization of SIGMA Model

**Figure 2:**
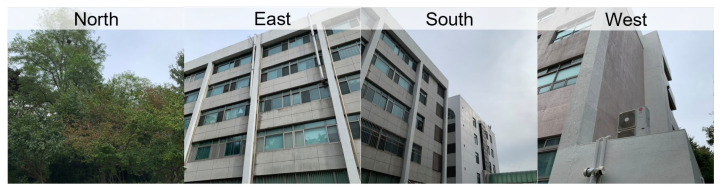
Urban environment used to analyze the characteristics of the SIGMA model.

The urban environment shown in Figure 2 was selected to analyze the characteristics of the SIGMA model. The northern side of the site is surrounded by a forested area, and the eastern, western, and southern sides are blocked by low-rise buildings. In this environment, signals from the east, west, and south can be blocked by buildings or affected by severe multipath. Because this environment is similar to an urban canyon, characteristics that differ from those observed in open areas can be identified.

6 h of observations were acquired on DOY 260 and 261 in 2025 using the static data collection method described in Section 2.1. To compute the code-pseudorange residuals, the reference station of Inha University (IHUB), located approximately 500 m away, was used. The total number of observations acquired was 160,000 for GPS L1, 70,000 for Galileo E1, 110,000 for BeiDou B2, 27,000 for GPS L5, and 44,000 for Galileo E5.

**Figure 3:**
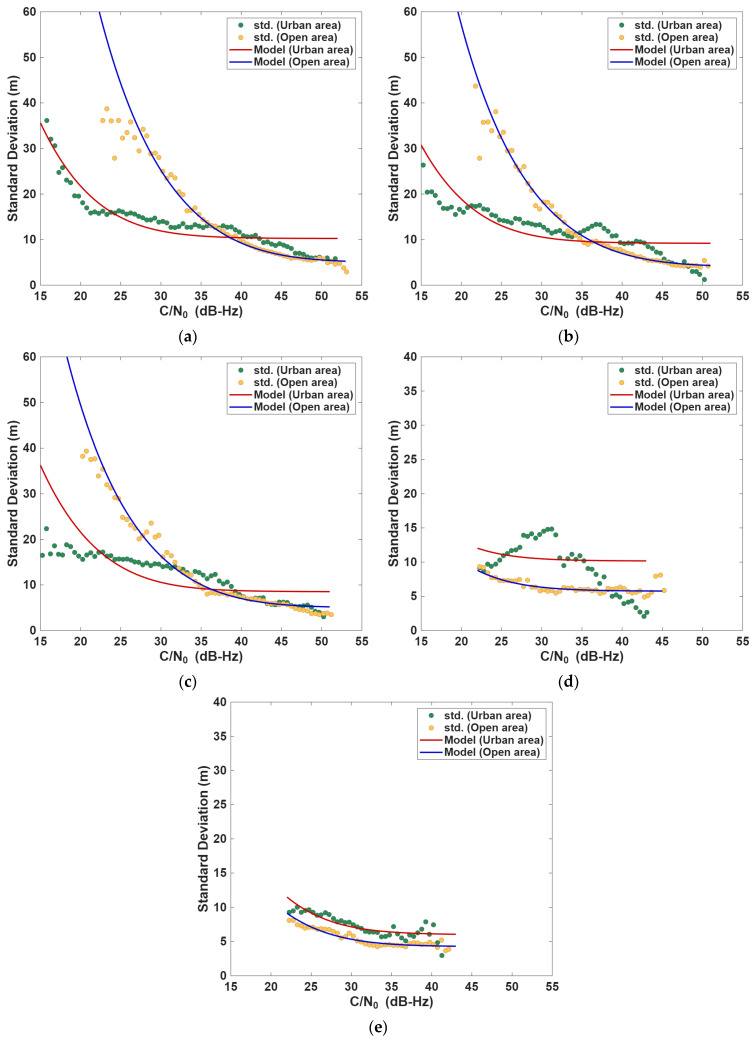
Comparison of SIGMA model fitting results for the standard deviation of code-pseudorange residuals between the urban and open-area environments for each signal: (**a**) GPS L1; (**b**) Galileo E1; (**c**) BeiDou B2; (**d**) GPS L5; (**e**) Galileo E5.

The double-differenced code-pseudorange residuals were computed using urban data, and the standard deviations for each bin were fitted using Equation (8). The fitted model was compared with an open-area model using the parameters listed in Table 2. As shown in Figure 3, the standard deviation and regression models in urban areas are indicated by green and red circles, respectively. In the open area, the deviation and regression models are denoted by yellow and blue circles, respectively.

Two characteristics were identified in this urban area: (1) model mismatch and (2) an elevated residual floor. Model mismatch was observed for GPS L1, GAL E1, BDS B2, and GPS L5. In particular, BDS B2 and GPS L5 exhibited residual patterns that differed substantially from those in open areas, indicating that the SIGMA model was not suitable. GPS L1 and GAL E1 exhibited different behaviors in open areas. Their residuals followed the SIGMA model only when C/N_0_ was below approximately 35 dB-Hz; above this threshold, the model no longer matched the standard deviations.

Second, an elevated residual floor was observed for GAL E5. In this case, the residuals followed the SIGMA model in both urban and open areas; however, the standard deviation was several meters higher in urban areas. This indicates that an additional covariance inflation term is needed under such conditions to account for the elevated residual floor. A similar pattern was observed for GPS L1 and BDS B2, particularly when C/N_0_ exceeded approximately 35 dB-Hz.

This analysis highlights the limitations of the SIGMA model in urban areas and indicates that an appropriate covariance model and additional covariance inflation are both required. In particular, signals with C/N_0_ values above approximately 35 dB-Hz exhibited larger residual deviations. Therefore, covariance inflation was introduced to construct adaptive observation covariance models. In addition, a C/N_0_ cutoff of 30 dB-Hz was adopted in the positioning algorithm to maintain the effectiveness of covariance inflation, as a 35 dB-Hz cutoff would significantly reduce the number of visible satellites.

### 3.2. Observation Covariance Inflation Using ECI-F

ECI-F was introduced to construct an inflation strategy for the SIGMA model. ECI-F quantifies relative observation quality with respect to open-area conditions. As ECI-F increases, the observation quality degrades, indicating that the observation environment approaches urban conditions. ECI-F is computed from C/N_0_ and PDOP [27], as shown in Equation (10).

(10) $$ECI-F=\gamma \left(W_{PDOP}I_{PDOP}+W_{C/N_{0}}I_{C/N_{0}}\right)\;I_{C/N_{0}}=\frac{1}{N}\sum_{i=1}^{N}\left|\left(C/N_{0}^{i}\left(t\right)-\mu_{ref}\right)/\sigma_{ref}\right|,\;I_{PDOP}=ln\left(PDOP\right)$$

$I_{C/N_{0}}$ represents the average of the normalized C/N_0_ values for all observed satellites. This metric is calculated using the reference mean ($\mu_{ref}$) and standard deviation ($\sigma_{ref}$) obtained under open-area conditions. Here, $C/N_{0}^{i}\left(t\right)$ denotes the C/N_0_ value of the i-th satellite observed at a specific epoch t, and N is the number of satellites used in the calculation. $I_{PDOP}$ is defined as the natural logarithm of PDOP to reduce the scale difference between PDOP and $I_{C/N_{0}}$, allowing both metrics to be combined in the ECI-F calculation. Both metrics are combined using the weights, $W_{PDOP}$ and $W_{C/N_{0}}$, and the scaling factor γ. The weights are calculated using empirically determined functions, while γ was set to 2 to adjust the scale of ECI-F.

Here, larger values of $I_{C/N_{0}}$ and $I_{PDOP}$ indicate a higher likelihood of degraded reception. Accordingly, a smaller ECI-F value indicates an environment closer to open-area conditions, and a larger value indicates a more challenging observation environment.

**Figure 4:**
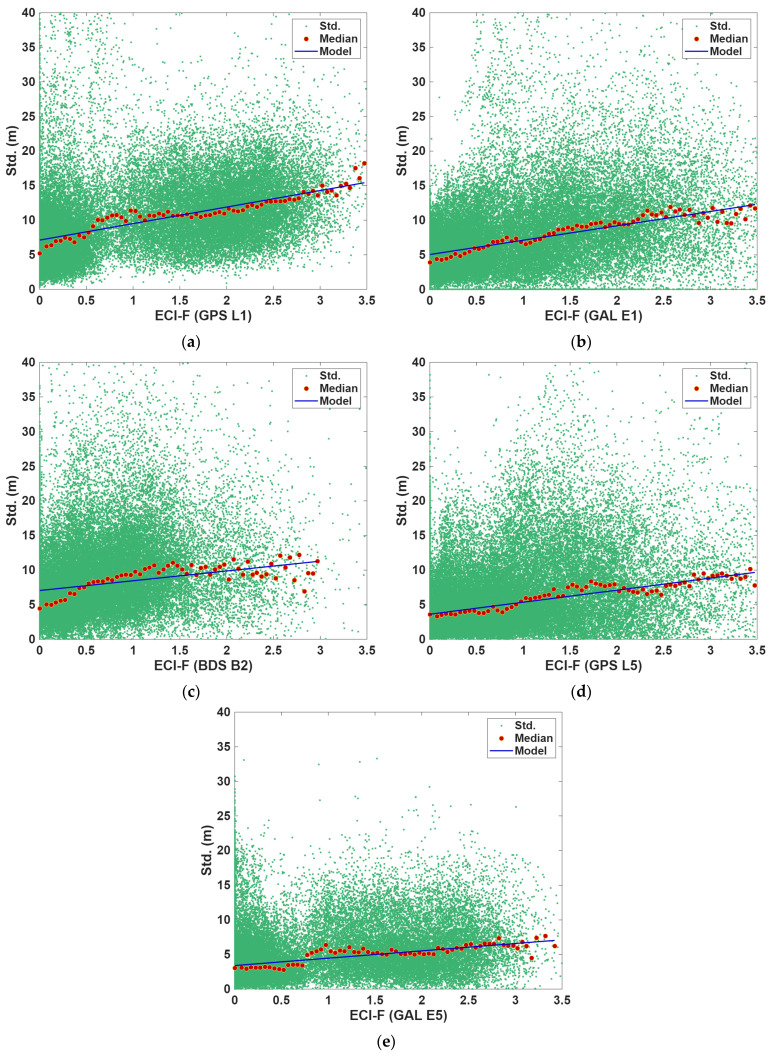
Relationship between ECI-F and the standard deviation of code-pseudorange residuals for each signal: (**a**) GPS L1; (**b**) Galileo E1; (**c**) BeiDou B2; (**d**) GPS L5; (**e**) Galileo E5. The green dots show the standard deviation values, the red circles show the median values for 0.05 ECI-F intervals, and the blue line shows the fitted linear regression model.

To construct an adaptive covariance model using ECI-F, we analyzed the relationship between ECI-F and code-pseudorange residuals. The residuals were computed from the same dataset used in Section 3.1. For each epoch and signal, ECI-F and the standard deviation of code-psuedorange were obtained, yielding one deviation for each epoch and signal. ECI-F values were then grouped into bins with an interval of 0.05, and the median of the standard deviations was calculated for each bin. A linear regression model was subsequently fitted to these bin-wise median values. The corresponding residual deviations (green dots), the median of residual deviations (red circles), and regression model (blue line) are shown in Figure 4.

Although the direct relationship between ECI-F and residual deviation was not clear, the median of standard deviation exhibited a linear trend. This indicates that the expected deviation of code-pseudorange residuals increases approximately linearly as ECI-F increases. Therefore, the relationship between ECI-F and the median of standard deviations can be modeled as a linear function.

As shown in Equation (11), the regression model comprises a bias $\left(a_{ECI-F}\right)$ and slope term $\left(b_{ECI-F}\right)$. The ECI-F regression model predicts changes in the median of the deviations using the regression coefficients and changes in ECI-F.

(11) $$Median\left(\sigma_{\rho }\right)=a_{ECI-F}+b_{ECI-F}\times \left(ECI-F\right)$$

The regression coefficients and corresponding coefficients of determination (R^2^) were obtained for each signal. As presented in Table 3, the R^2^ exceeds 0.80 for GPS L1/L5 and GAL E1/E5, whereas BDS B2 shows a lower value of approximately 0.67. Compared with the other signals, this value is 0.16 lower than that of GAL E1, 0.19 lower than that of GPS L5 and GAL E5, and 0.20 lower than that of GPS L1.

The inflation factor α in Equation (12) was derived from the ECI-F regression model by removing the bias term, adding 1 so that α=1 under ideal open-area conditions, and squaring the term to match the variance unit. This allows the SIGMA-based variance to be inflated according to the environmental degradation represented by ECI-F.

(12) $$\alpha ={\left(Median\left(\sigma_{\rho }\right)-a_{ECI-F}+1\right)}^{2}\;R_{ECI-F}=\alpha \times \left(a+b\times 10^{-\left(C/N_{0}\right)/10}\right)$$

In the Kalman filter, a larger inflation factor increases the corresponding observation covariance, reduces the Kalman gain in Equation (7), and weakens the contribution of degraded observations to the coordinate update. Conversely, when the inflation factor decreases, its contribution increases or returns to the level of the original SIGMA model. In addition, this indicates that the environmental changes and observation-quality variations represented by ECI-F are numerically reflected in the observation covariance at each epoch, enabling adaptive covariance adjustment over time.

### 3.3. NLOS Satellite Detection and Mitigation

ECI-F evaluates environmental changes and inflates the observation covariance for each signal type. However, in urban environments, NLOS satellites must be considered separately because the ECI-F-based adaptive covariance model does not directly address NLOS effects. NLOS signals may exhibit high C/N_0_ values because of severe multipaths [43], which may increase their contribution to ECI-F calculations. Conversely, signals with low C/N_0_ and small residuals may also increase ECI-F, leading to a larger inflation factor unnecessarily. Therefore, an additional strategy is required to detect NLOS satellites and mitigate their effects. To detect NLOS satellites, the normalized C/N_0_ metric of the i-th satellite ($I_{C/N_{0}}^{i}$), and the normalized code-pseudorange residual metric of the i-th satellite ($I_{Res_{\rho }}^{i}$), were introduced, as expressed in Equation (13). Here, $I_{Res_{\rho }}^{i}$ is calculated from the code-pseudorange residual (${Res}_{\rho }^{i}$), which represents the code-pseudorange residual of the i-th satellite. Because both metrics are derived from open-area statistics ($\mu_{OA,C/N_{0}}$, $\sigma_{OA,C/N_{0}},\;\mu_{OA,Res_{\rho }},\;\sigma_{OA,Res_{\rho }}$), they increase when the signal reception condition becomes degraded relative to open-area conditions.

(13) $$\begin{array}{cc} I_{C/N_{0}}^{i} & =\left|\left(C/N_{0}^{i}\left(t\right)-\mu_{OA,C/N_{0}}\right)/\sigma_{OA,C/N_{0}}\right|\\ I_{Res_{\rho }}^{i} & =\left|\left(Res_{\rho }^{i}\left(t\right)-\mu_{OA,Res_{\rho }}\right)/\sigma_{OA,Res_{\rho }}\right| \end{array}$$

Two types of multipath-contaminated signals were identified, namely high C/N_0_ with large residuals (Type 1) and low C/N_0_ with small residuals (Type 2). As expressed in Equation (14), a two-sigma rule is used to identify observations outside the central approximately 95% variation range of the open-area residual distribution as abnormal observations. Signals with $I_{C/N_{0}}^{i}<2$ and $I_{Res_{\rho }}^{i}>2$ were classified as Type 1 and their covariances were heavily inflated by a factor of $10^{6}$, thereby reducing their contributions to the coordinate update.

(14) $$\left\{\begin{array}{c} \alpha^{i}=10^{6}\;I_{C/N_{0}}^{i}<2\;and\;I_{Res_{\rho }}^{i}>2\;\;(Type\;1)\\ \alpha^{i}=1\;\;\;\;\;I_{C/N_{0}}^{i}>2\;and\;I_{Res_{\rho }}^{i}<2\;\;\left(Type\;2\right)\\ \alpha^{i}=1\;\;\;\;\;\;otherwise\;\;\;\;\;\;\;\;\;\;\;\;\left(Others\right) \end{array}\right.$$

By contrast, signals with $I_{C/N_{0}}^{i}>2$ and $I_{Res_{\rho }}^{i}<2$ were classified as Type 2. No additional inflation was applied to these signals because their effects on the positioning filter were less severe than those of the Type 1 signals. Notably, Type 2 signals may correspond to attenuated LOS signals. However, because this pattern can also occur under NLOS conditions [44], they are classified as NLOS signals in this study. Signals satisfying $I_{C/N_{0}}^{i}<2$ and $I_{Res_{\rho }}^{i}<2$, or $I_{C/N_{0}}^{i}>2$ and $I_{Res_{\rho }}^{i}>2$, were categorized as “Others”. Unlike Type 1 and Type 2 signals, these signals exhibit a relatively consistent relationship between C/N_0_ and code-pseudorange residuals and were therefore not classified as NLOS signals. For these signals, no additional NLOS-based covariance inflation was applied; instead, only the ECI-F-based covariance inflation was adopted. This additional inflation procedure was applied before calculating the ECI-F, thereby preventing NLOS satellite effects from influencing the corresponding ECI-F-based covariance inflation model.

This method detects NLOS satellites using normalized metrics derived from open-area statistics. Therefore, the two-sigma rule was used to compare the relative likelihood of a given observation with respect to open-area conditions. For Type 1 signals, the signal strength remains within a range that is commonly observed in open areas, whereas the residual magnitude does not. This implies that, although the C/N_0_ value is consistent with open-area conditions, the corresponding residual is unusually large relative to open-area statistics, indicating the presence of strong multipaths or NLOS effects.

**Figure 5:**
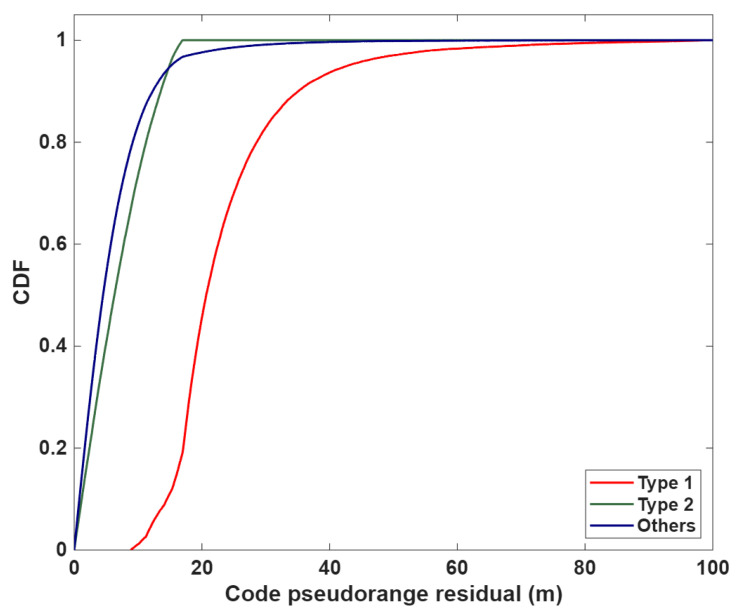
Cumulative distribution functions (CDFs) of code-pseudorange residuals for satellites classified by the proposed non-line-of-sight NLOS detection scheme: Type 1, Type 2, and Others. Type 1, Type 2, and Others are shown by the red, green, and blue solid lines, respectively.

To illustrate the NLOS satellite detection method, Equation (14) is applied to data collected from the environment shown in Figure 2. The resulting classifications and code-pseudorange residuals are shown in Figure 5 for three groups: Type 1, Type 2, and “Others”, which refers to signals that were classified as neither Type 1 nor Type 2. The residual magnitudes of the “Others” and Type 2 groups are similar. However, a slight difference is observed in the 50th percentile: the 50th percentile of the “Others” and Type 2 groups is 4.51 and 6.30 m, respectively. This is because Type 2 signals generally exhibit lower C/N_0_ values than the “Others” group.

By contrast, the Type 1 group exhibits substantially larger code-pseudorange errors than the “Others” and Type 2 groups. The 50th percentile error of Type 1 is 20.66 m, which is approximately 15 m larger than the 50th percentile of the other two groups. This implies that such observations may cause the positioning filter to diverge because their covariance can be underestimated relative to their actual error magnitude when the SIMGA model is used. Furthermore, in harsh environments, the impact of Type 1 satellites on positioning accuracy may become more severe. Therefore, the influence of Type 1 satellites should be significantly reduced to prevent large positioning errors, as expressed in Equation (13).

## 4. Experiments and Results

This section presents an evaluation of the proposed method in open-area, semi-urban, and urban-canyon environments. Section 4.1 summarizes the validation datasets. Section 4.2 presents the results of the open-area and semi-urban experiments, and Section 4.3 provides the results of urban-canyon and further analysis.

### 4.1. Datasets and Test Scenarios

**Figure 6:**
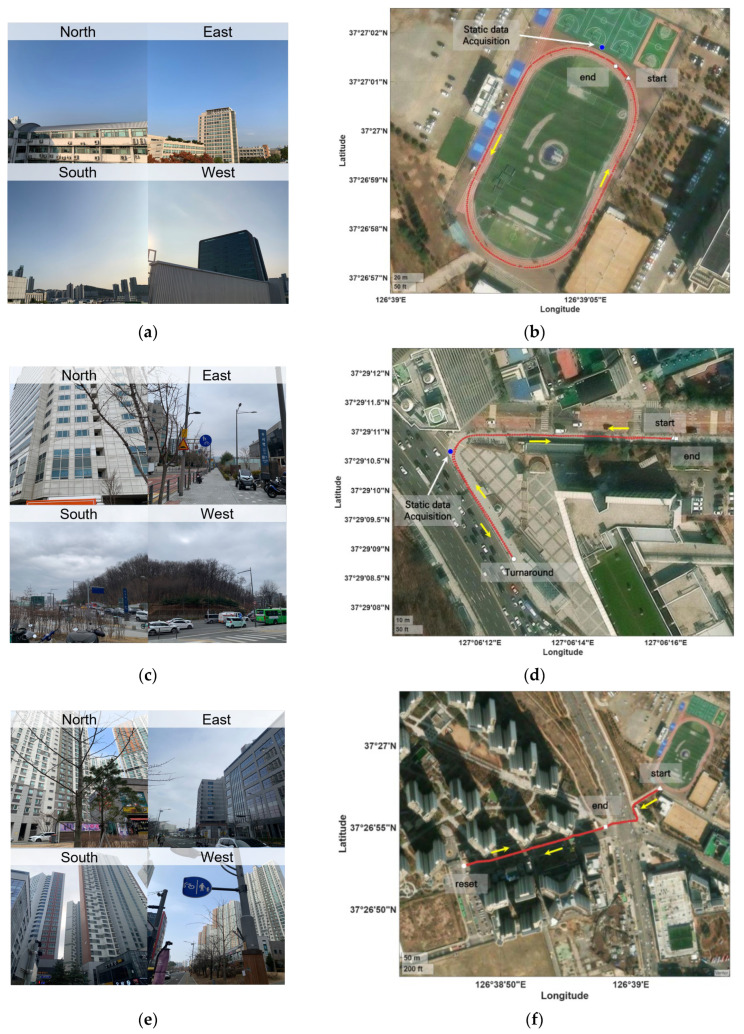
Test environments and routes used in the kinematic experiments: (**a**) open-area environment (T1); (**b**) test route for T1; (**c**) semi-urban environment (T2); (**d**) test route for T2; (**e**) urban-canyon environment (T3); (**f**) test route for T3. In (**b**,**d**,**f**), the white triangles, squares, and circles denote the start, end, and turnaround points, respectively. The red lines and yellow arrows indicate the test route and direction of travel, respectively, while the blue circles denote the static data acquisition locations.

Test 1 (T1) was conducted on the athletic field at Inha University, which is close to an open-area environment. At this site, as shown in Figure 6a, multipath effects can occur at low elevations on the western side, whereas the other sides offer clear satellite visibility. As shown in Figure 6b, a kinematic test was conducted by walking along a track (red line) from the starting point (white triangle) to the endpoint (white circle). In addition, static data were collected near the track (blue circles).

Test 2 (T2) was conducted at a subway station in Seoul, which represents a semi-urban environment. As shown in Figure 6c, signals from the north and east sides may be affected by multipath effects, whereas the west and south sides have a relatively clear sky view. As shown in Figure 6d, the kinematic test began at the starting point (white triangle) and followed the sidewalk. After reaching the turnaround point (white circles), the trajectory returned to the endpoint (white squares). During data collection, high-rise buildings on the northern side were expected to affect observation quality. Static data were collected in front of the building on the northern side (blue circle).

Test 3 (T3) was conducted in an urban canyon. As shown in Figure 6e, high-rise buildings are located toward the east and west sides, probably causing severe multipaths in the north and south directions. Kinematic data was collected along the trajectory, as shown in Figure 6f. The test began at the starting point (white triangle) and continued along the sidewalk until the reset point (white circle). At this point, the logger was restarted, and data collection continued until the endpoint (white square). In this test, the logger was restarted to examine the performance of the proposed method during initial convergence in a harsh environment.

For T1 and T2, multiple datasets were collected to examine the repeatability and robustness of the proposed method under various observation conditions. As presented in Table 4, each static test lasted 300 s, and 18 and 24 datasets were collected for T1 and T2, respectively. However, the baseline length differed substantially between the two sites: T1 used the IHUB reference station as the base station, resulting in a short baseline of approximately 0.3 km, whereas T2 used the PPSOL Inc. reference station (PPHQ) as the base station, resulting in a relatively long baseline of approximately 20 km. For the kinematic test, five datasets were collected for T1 and 15 for T2. The average test duration was approximately 280 s for T1 and 240 s for T2, with corresponding average walking speeds of 1.05 and 0.86 m/s, respectively.

In T3, only one dataset was collected to analyze the proposed method in detail. The baseline was approximately 0.7 km from the reference station of IHUB. The test duration was 539 s, with an average velocity of approximately 1.22 m/s. The performance was evaluated using circular error probable (CEP) and distance root mean square (DRMS).

### 4.2. Validation in Open-Area and Semi-Urban Environments

**Figure 7:**
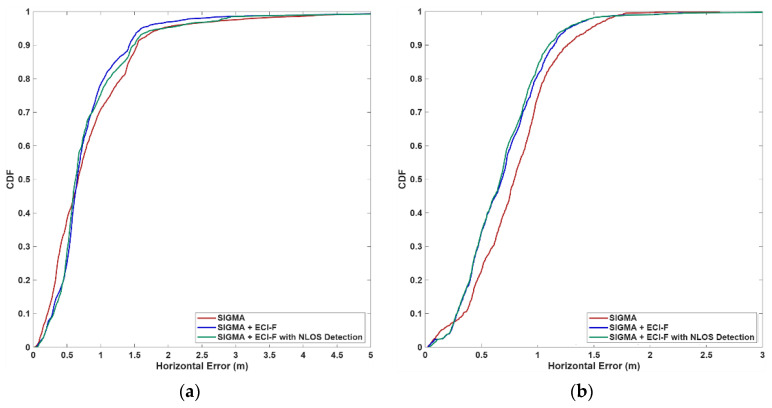
CDFs of horizontal positioning errors for the open-area static and kinematic datasets: (**a**) static; (**b**) kinematic. The red, blue, and green lines represent the results obtained using the SIGMA model, SIGMA model with ECI-based covariance inflation, and SIGMA model with both ECI-F and NLOS detection, respectively.

This subsection evaluates the proposed method in the open-area (T1) and semi-urban (T2) environments. In the open-area test, the proposed method improved horizontal accuracy, although only a small inflation factor was expected in this environment. As shown in Figure 7, the conventional SIGMA model (red line) was improved by applying ECI-F (blue line) and ECI-F with NLOS detection (green line) in both static and kinematic tests. In the static test, the 50th percentile remained unchanged, the higher-percentile errors decreased, and the lower-percentile errors slightly increased. In the kinematic test, most percentile errors decreased.

These results show that ECI-F varied in both the static and kinematic tests even in an open-area environment. A possible explanation is that the buildings on the western side of the test site, as shown in Figure 6a, degraded the observation quality. In addition, in the kinematic test, the distance between the participant and surrounding buildings changed as the participant moved along the test route, probably causing further variation in observation quality.

**Figure 8:**
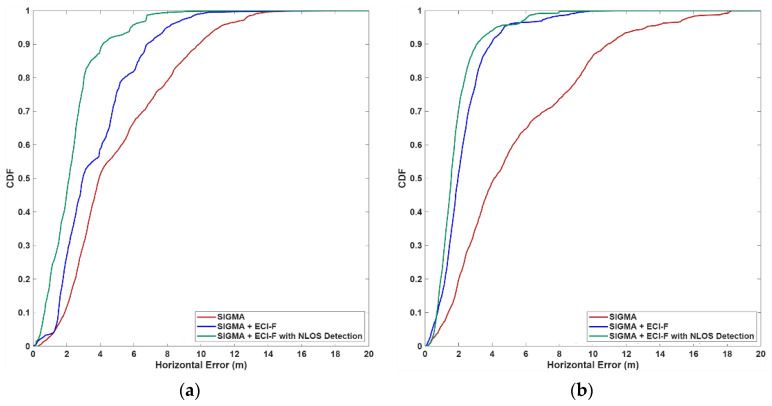
CDFs of horizontal positioning errors for the semi-urban static and kinematic datasets: (**a**) static; (**b**) kinematic. The red, blue, and green lines represent the results obtained using the SIGMA model, SIGMA model with ECI-based covariance inflation, and SIGMA model with both ECI-F and NLOS detection, respectively.

The proposed method exhibited a larger improvement in CEP in the semi-urban test than in the open-area test. As shown in Figure 8, the SIGMA model (red line) exhibited degraded accuracy in both the static and kinematic tests compared with the open-area test. However, applying ECI-F (blue line) and ECI-F with NLOS detection (green line) improved accuracy by several meters. In particular, the additional improvement provided by NLOS detection was more evident than that in the open-area test. This indicates that NLOS satellites are observed more frequently in semi-urban environments and contribute to the degraded performance of the SIGMA model. Furthermore, the improvement was greater in the kinematic test than in the static test. This is because high-rise buildings on the northern side of the test site can cause larger temporal variations in observation quality as the participant moved along the test route, as shown in Figure 6c.

Compared with the results in Figure 7, gross errors above the 90th percentile were substantially reduced, as shown in Figure 8. This indicates that the proposed method effectively controls the observation covariance by evaluating observation quality and detecting NLOS satellites.

As presented in Table 5, the proposed method achieves submeter accuracy in an open-area environment and meter-level accuracy in a semi-urban environment. In the open-area test, the SIGMA model exhibited submeter-level CEP50 in both the static and kinematic cases, and CEP95 ranged from approximately 1 to 2 m. In addition, DRMS was at the meter level in the static case, whereas the kinematic case exhibited submeter-level precision. By contrast, both accuracy and precision degraded in the semi-urban environment, where CEP and DRMS ranged from approximately 3 to 12 m. In particular, CEP95 increased to approximately 10–12 m, indicating poorer accuracy.

In the open-area test, the proposed method achieved submeter-level horizontal accuracy based on CEP50, with values of 0.65 m and 0.68 m for the static and kinematic tests, respectively. Since CEP50 represents the median horizontal error, these results indicate that the positioning accuracy reached the several-decimeter level.

In the semi-urban test, CEP and DRMS were improved by several meters. In particular, the combination of the ECI-F-based inflation model and NLOS detection showed the greatest improvement in the semi-urban environment. A greater improvement in the semi-urban area indicates that ECI-F increases as observation quality degrades, resulting in a larger inflation factor. In addition, NLOS satellites are observed more frequently because of signal reflection and scattering in the semi-urban environment, leading to further covariance inflation.

### 4.3. Additional Validation in Urban Canyon: Single-Session Case Study

**Figure 9:**
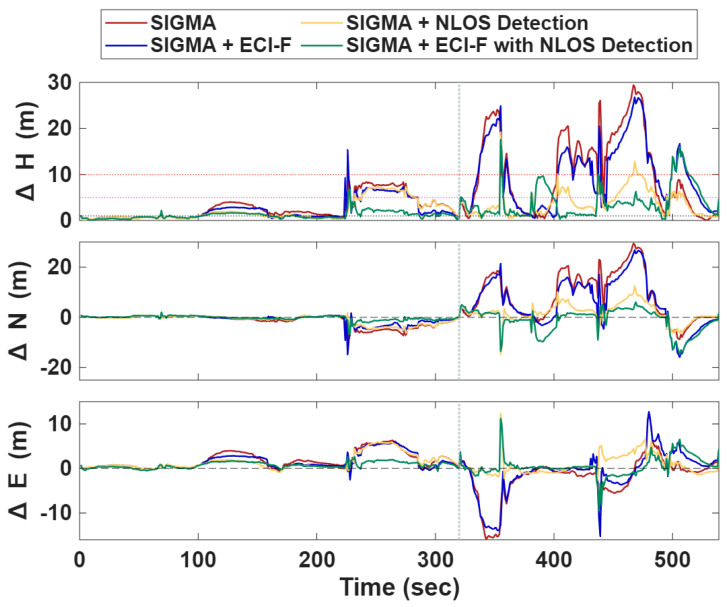
Time series of position errors in the urban canyon environment for different observation covariance adjustment methods. The red, blue, yellow, and green lines denote SIGMA, SIGMA + ECI-F, SIGMA + NLOS detection, and SIGMA + ECI-F with NLOS detection, respectively. In the top panel, the dashed horizontal lines indicate the LBS accuracy requirements of 1, 5, and 10 m, and the gray dashed lines at 320 s indicate the logger restart period.

This subsection describes the evaluation of the proposed method in the urban-canyon environment (T3). In this test, the position estimates diverged frequently because of severe multipath effects. As shown in Figure 9, the error of the SIGMA model (red line) begins to increase to approximately 10 m before reaching the reset point (gray dashed line). After the reset point, the position estimates diverge by approximately 30 m, and this divergence pattern occurs repeatedly until the end of the test. This indicates that the reset point shown in Figure 6f is particularly vulnerable to multipath effects, whereas the starting point has a relatively clear sky view.

In this environment, the proposed method improves positioning accuracy. In particular, the ECI-F-based inflation method (blue line) improves accuracy by several meters, and the combination of ECI-F and NLOS detection (green line) reduces gross errors by approximately tens of meters. This improvement is mainly attributed to the NLOS detection results (yellow line), which significantly reduce the divergence after the reset point. Although the ECI-F-based inflation method reduces the error magnitude, it exhibits a pattern similar to that of the SIGMA model. By contrast, the method combining ECI-F with NLOS detection does not follow this error pattern, indicating that the divergence observed in the SIGMA model is mainly caused by NLOS satellites.

The ECI-based inflation method and NLOS detection also exhibit complementary behavior. Before reaching the reset point, the SIGMA model diverges by tens of meters after approximately 225 s. During this period, the ECI-F-based inflation and NLOS detection methods are not sufficiently effective when applied separately. However, their combined use reduces the errors more effectively. This implies that applying the two methods separately may lead to inadequate covariance adjustment and limited improvement. Furthermore, NLOS detection enhances the ECI-F-based inflation model by reducing divergence patterns. This is because the ECI-F-based inflation model alone does not explicitly account for NLOS satellites.

As presented in Table 6, the proposed method significantly improves the SIGMA model. By applying ECI-F-based inflation, CEP50, CEP95, and DRMS improved by 14.23%, 34.30%, and 8.87%, respectively. Furthermore, NLOS detection significantly improves CEP and DRMS, resulting in improvements of 51.32%, 65.38%, and 58.58% in CEP50, CEP95, and DRMS, respectively.

These results can be interpreted from the perspective of LBS applications. In LBS, horizontal accuracy requirements can be divided into three categories: (1) high (dH < 1 m), (2) medium (1 m < dH < 5 m), and (3) low accuracy (5 m < dH < 10 m). Among GNSS-based applications, navigation generally requires at least medium accuracy [2]. In the urban-canyon test, the proposed method achieves high accuracy for 36.17% of the test duration and medium accuracy for 51.20%. This indicates that 87.37% of the test duration satisfies at least the medium-accuracy requirement, whereas the SIGMA model achieves only 64.18%. In addition, the proposed method reduces the proportion of low-accuracy solutions by 7.79%, indicating that the position estimates become more reliable.

**Figure 10:**
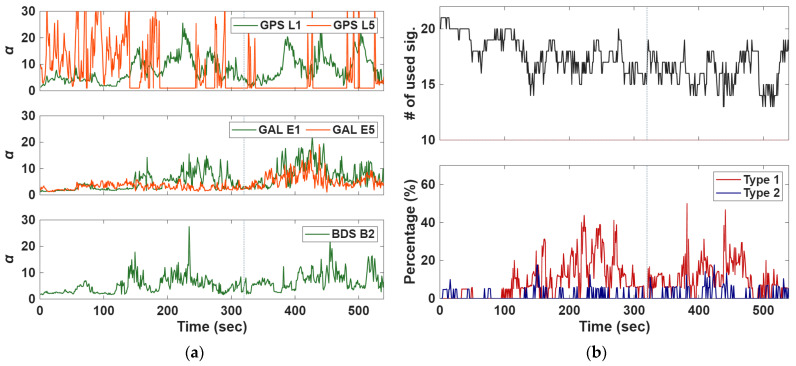
Time series of the ECI-based scaling factors and NLOS detection results in the urban canyon test: (**a**) ECI-based inflation factors for GPS L1/L5, Galileo E1/E5, and BeiDou B2; (**b**) number of used observations and percentage of satellites classified as Type 1 and Type 2. The black line in (**b**) indicates the number of used observations, and the red and blue lines indicate the percentages of Type 1 and Type 2 satellites, respectively. The gray dashed lines indicate the logger restart epochs.

To explain this improvement, the inflation factors of the ECI-F-based method and NLOS satellite detection results are shown in Figure 10. In Figure 10a, GPS L5 exhibits frequent fluctuations in the inflation factor, indicating that the observation quality of GPS L5 varies rapidly over time. Except for GPS L5, the other signals exhibit patterns similar to the position error pattern shown in Figure 9. During the first 100 s, the inflation factors vary slowly; afterward, they increase and fluctuate as the participant walks through the urban canyon. In addition, the inflation factors vary across signal types, implying different contributions to the position estimates. In particular, GAL E5 exhibits smaller fluctuations and a lower inflation magnitude than GAL E1, indicating that GAL E5 observations substantially influence coordinate updates.

As shown in Figure 10b, the number of signals used varies rapidly during the test because high-rise buildings can block signals, and extremely degraded signals can be excluded. After 100 s, Type 1 signals are detected frequently, and occasionally, 40–50% of the observations are classified as Type 1. This indicates that the observations used in this test were strongly degraded by severe multipath effects, which contributed to the error growth shown in Figure 9. In addition, because the covariances of Type 1 signals were substantially inflated, the resulting gross errors were reduced. Type 2 signals were detected only occasionally, and their proportion was lower than that of Type 1 signals. This indicates that Type 1 signals had a more significant effect in the urban environment.

In this test, NLOS satellites were detected, and their corresponding observation covariances were inflated. Figure 9 and Figure 10 show that NLOS-contaminated observations are a major cause of coordinate divergence. Therefore, an additional analysis was conducted to evaluate whether the detected NLOS satellites were blocked by nearby buildings. For this analysis, two timestamps of 162 and 235 s were selected. At these times, the participant was walking along the sidewalk in the urban canyon, where the effects of high-rise buildings could be clearly evaluated. As no 3D building database was available for the test site, the surrounding building envelope was approximated manually. Specifically, the distance between the receiver and building exterior was measured, and the building height was estimated by assuming a floor height of 3 m. This procedure was conducted at least 10 points, and the corresponding azimuth and elevation angles were computed.

**Figure 11:**
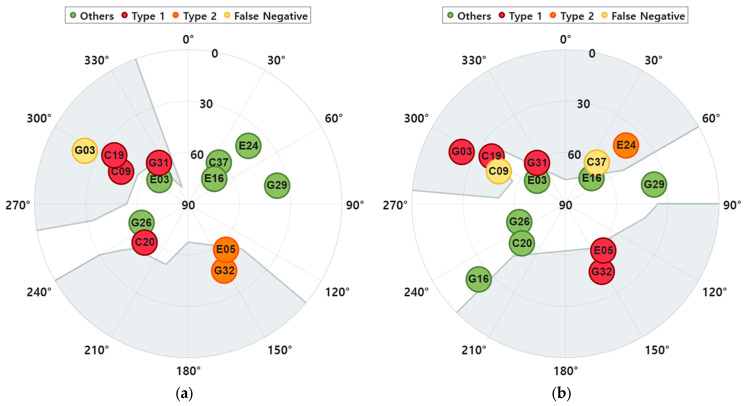
Satellite skyplots and building outlines during the kinematic test: (**a**) at 162 s; (**b**) at 235 s. The gray shaded regions indicate areas obscured by buildings. The green, red, and orange circles denote satellites classified as LOS, Type 1 NLOS, and Type 2 NLOS, respectively, and the yellow circles denote false negatives, i.e., satellites obscured by buildings but classified as LOS.

As shown in Figure 11a, the northeastern and southern sides were blocked at 162 s. In the blockage region (gray patch), Type 1 (red circle) and Type 2 (orange circle) signals were detected. These signals were expected to be affected by severe multipath effects; therefore, their corresponding covariances were inflated. However, a false negative occurred for GPS PRN 3, although this satellite was at a low elevation and blocked by buildings. Furthermore, BDS PRN 20 was ambiguous because of uncertainty in the building envelope. This ambiguous status is partly related to the uncertainty in manually estimated building heights. In this study, building heights were manually estimated by assuming an average floor height of 3 m because a detailed 3D building database was unavailable. This manual estimation may include errors due to roof structures, variations in floor height among buildings, and higher floor heights in commercial buildings.

As the participant moved toward the central part of the urban canyon, the surrounding building envelope changed. As shown in Figure 11b, at 235 s, most of the north and south sides were blocked; therefore, satellites from these directions were more likely to be obstructed. Galileo PRN 24 was classified as Type 2, whereas it was classified as Others at 162 s. In addition, Galileo PRN 5, GPS PRN 32, and GPS PRN 3 were changed to Type 1. These results show that signal blockage conditions changed as the participant moved and that the proposed method could detect the corresponding blocked signals.

## 5. Discussion

In this study, we observed a linear relationship between ECI-F and the standard deviation of the code-pseudorange residuals. Based on this relationship, we established a linear regression model to adjust the observation covariance for each signal type. Because ECI-F represents relative observation quality with respect to open-area conditions, the model inflates the covariance of signal types observed in harsh environments. However, the ECI-F-based regression model should be separately optimized for different device types because measurement quality varies across devices. Nevertheless, this regression model can be applied to both relative and single-point positioning because ECI-F is computed without differencing. However, the proposed regression model achieved coefficients of determination ranging from 60% to 80%, depending on the signal type. This indicates that the observation covariance of some signal types may have been inaccurately inflated. In particular, BDS B2 showed the lowest R^2^ value of 0.67. Although the number of BDS B2 observations was comparable to those of the other signal types, low-quality observations were relatively less represented. This imbalance may have weakened the linear relationship between ECI-F and the standard deviation of code-pseudorange residuals. With more BDS B2 data collected under degraded environments, the regression model is expected to become more accurate.

Furthermore, we demonstrated that normalized C/N_0_ and code-pseudorange residuals can be used to detect NLOS satellites in urban canyons. Because these two metrics are derived from open-area statistics, they also reflect environmental degradation relative to open-area conditions, similar to ECI-F. This detection method uses a two-sigma rule to identify NLOS satellites, and their observations are assigned lower weights to reduce the impact of multipath effects. These results show that contextual information can enhance the robustness of positioning filter. In future work, further improvements may be achieved by selecting appropriate detection thresholds and covariance inflation factors.

Although T2 showed improvement in positioning accuracy under an approximately 20 km baseline condition, long-baseline RTK can still be affected by residual ionospheric and tropospheric errors that are not fully eliminated by double differencing. These remaining atmospheric residuals may influence the code-pseudorange residuals used for NLOS satellite detection and lead to inaccurate classification. Therefore, the proposed method should be applied with caution in long-baseline RTK.

## 6. Conclusions

This study proposed an adaptive observation covariance model based on ECI-F and NLOS satellite detection to improve smartphone RTK positioning accuracy. The proposed methods were evaluated in open-area, semi-urban, and urban-canyon environments. Experimental results showed that the proposed method improved CEP by several tens of centimeters in both static and kinematic cases in open areas. In urban areas, CEP95, CEP50, and DRMS improved by several meters to tens of meters. In particular, the additional use of NLOS satellite detection was effective in reducing gross positioning errors in urban-canyon environments, where severe multipath and signal blockage frequently degrade the observation quality. These results show that contextual information can be applied to adjust the observation covariance, thereby improving positioning accuracy and precision. Moreover, ECI-F represents environmental quality as a continuous indicator rather than a categorical class, allowing the stochastic model to respond more flexibly to environmental changes. In addition, the results showed that the adaptive observation covariance model using ECI-F and NLOS detection could also enhance the robustness of position estimation. In terms of practical implementation, the proposed method relies on a pre-defined covariance model and rule-based NLOS detection, requiring only simple epoch-wise calculations. Therefore, it is computationally efficient and suitable for real-time smartphone GNSS positioning.

## Figures and Tables

**Table 1:** Smartphone and logging configuration.

Category	Specification
Device	Samsung Galaxy S21+ (Samsung Electronics Co., Ltd., Suwon, Republic of Korea)
OS version	Android 14
SoC/CPU	Exynos 2100 (1 × Cortex-X1 ~2.9 GHz + 3 × A78 ~2.8 GHz + 4 × A55 ~2.2 GHz)
GPU/RAM	Mali-G78 MP14, 8 GB RAM
GNSS constellation	GPS, Galileo, BeiDou, GLONASS, QZSS, NAVIC, SBAS
GNSS signals	L1/E1/B2/G1 + L5/E5a
Logging app	Custom logger

**Table 2:** SIGMA model parameters and carrier-to-code noise ratios for each constellation and frequency.

Constellation/Frequency	a	b	$\sigma_{\Phi }^{2}:\sigma_{\rho }^{2}$
GPS/L1	24.59	611,342	1:(449)^2^
GAL/E1	16.09	324,222	1:(453)^2^
BDS/B2	24.91	241,787	1:(520)^2^
GPS/L5	33.06	7005	1:(83)^2^
GAL/E5	18.15	10,370	1:(85)^2^

**Table 3:** Estimated ECI-F regression parameters ($a_{ECI-F}$ and $b_{ECI-F}$) and coefficient of determination (R^2^) for each constellation and frequency.

Constellation/Freq.	$a_{ECI-F}$	$b_{ECI-F}$	R^2^
GPS/L1	5.95	1.60	0.87
GAL/E1	5.17	1.66	0.83
BDS/B2	6.44	1.00	0.67
GPS/L5	3.27	1.36	0.86
GAL/E5	3.11	1.17	0.86

**Table 4:** Summary of the static and kinematic test datasets.

Type	Baseline Length (km)	Number of Sets	Duration (s)	Velocity (m/s)
T1 (static)	≈0.3	18	300	0
T2 (static)	≈20	24	300	0
T1 (kinematic)	≈0.3	5	≈280	≈1.05
T2 (kinematic)	≈20	15	≈240	≈0.86
T3 (kinematic)	≈0.7	1	539	≈1.22

**Table 5:** Comparison of horizontal positioning accuracy and precision for the static and kinematic tests under different positioning methods.

Type	Type	CEP95 (m)	CEP50 (m)	DRMS (m)
SIGMA	T1 (static)	2.02	0.69	1.19
	T1 (kinematic)	1.47	0.79	0.58
	T2 (static)	10.91	3.50	4.27
	T2 (kinematic)	12.97	4.00	6.37
SIGMA + ECI-F	T1 (static)	1.72	0.66	1.19
	T1 (kinematic)	1.27	0.68	0.69
	T2 (static)	7.86	2.60	3.27
	T2 (kinematic)	4.65	1.94	2.52
SIGMA + ECI-F + NLOS Detection	T1 (static)	2.17	0.65	1.22
	T1 (kinematic)	1.26	0.68	0.69
	T2 (static)	5.82	2.14	2.75
	T2 (kinematic)	4.12	1.57	2.10

**Table 6:** Horizontal positioning accuracy statistics and relative improvement rates compared with the SIGMA model.

Type	CEP95 (m)	CEP50 (m)	DRMS (m)	dH < 1 (%)	1 < dH < 5 (%)	5 < dH < 10 (%)
SIGMA	22.62	3.02	8.79	25.41	38.77	16.69
SIGMA + ECI-F	14.86 (+34.30%)	2.59 (+14.23%)	8.01 (+8.87%)	29.31 (+4.10%)	34.50 (−4.27%)	16.14 (−0.55%)
SIGMA + NLOS Detection	7.83 (+65.38%)	1.74 (+42.38%)	3.70 (+57.90%)	33.95 (+8.54%)	45.26 (+6.49%)	18.36 (−1.67%)
SIGMA + ECI-F + NLOS Detection	7.25 (+67.94%)	1.47 (+51.32%)	3.64 (+58.58%)	36.17 (+10.76%)	51.20 (+12.43%)	8.90 (−7.79%)

## Data Availability

The raw data supporting the conclusions of this study will be made available by the corresponding author on request.
